# Chronic aryl hydrocarbon receptor activity impairs muscle mitochondrial function with tobacco smoking

**DOI:** 10.1002/jcsm.13439

**Published:** 2024-02-09

**Authors:** Liam F. Fitzgerald, Jacob Lackey, Ahmad Moussa, Sohan V. Shah, Ana Maria Castellanos, Shawn Khan, Martin Schonk, Trace Thome, Zachary R. Salyers, Nishka Jakkidi, Kyoungrae Kim, Qingping Yang, Russell T. Hepple, Terence E. Ryan

**Affiliations:** ^1^ Department of Physical Therapy University of Florida Gainesville FL USA; ^2^ Department of Applied Physiology and Kinesiology University of Florida Gainesville FL USA; ^3^ Myology Institute University of Florida Gainesville FL USA; ^4^ Center for Exercise Science, University of Florida Gainesville FL USA

**Keywords:** Atrophy, Cigarette, Dioxin, Skeletal muscle

## Abstract

**Background:**

Accumulating evidence has demonstrated that chronic tobacco smoking directly contributes to skeletal muscle dysfunction independent of its pathological impact to the cardiorespiratory systems. The mechanisms underlying tobacco smoke toxicity in skeletal muscle are not fully resolved. In this study, the role of the aryl hydrocarbon receptor (AHR), a transcription factor known to be activated with tobacco smoke, was investigated.

**Methods:**

AHR related gene (mRNA) expression was quantified in skeletal muscle from adult controls and patients with chronic obstructive pulmonary disease (COPD), as well as mice with and without cigarette smoke exposure. Utilizing both skeletal muscle‐specific AHR knockout mice exposed to chronic repeated (5 days per week for 16 weeks) cigarette smoke and skeletal muscle‐specific expression of a constitutively active mutant AHR in healthy mice, a battery of assessments interrogating muscle size, contractile function, mitochondrial energetics, and RNA sequencing were employed.

**Results:**

Skeletal muscle from COPD patients (*N* = 79, age = 67.0 ± 8.4 years) had higher levels of *AHR* (*P* = 0.0451) and *CYP1B1* (*P* < 0.0001) compared to healthy adult controls (*N* = 16, age = 66.5 ± 6.5 years). Mice exposed to cigarette smoke displayed higher expression of *Ahr* (*P* = 0.008), *Cyp1b1* (*P* < 0.0001), and *Cyp1a1* (*P* < 0.0001) in skeletal muscle compared to air controls. Cigarette smoke exposure was found to impair skeletal muscle mitochondrial oxidative phosphorylation by ~50% in littermate controls (Treatment effect, *P* < 0.001), which was attenuated by deletion of the AHR in muscle in male (*P* = 0.001), but not female, mice (*P* = 0.37), indicating there are sex‐dependent pathological effects of smoking‐induced AHR activation in skeletal muscle. Viral mediated expression of a constitutively active mutant AHR in the muscle of healthy mice recapitulated the effects of cigarette smoking by decreasing muscle mitochondrial oxidative phosphorylation by ~40% (*P* = 0.003).

**Conclusions:**

These findings provide evidence linking chronic AHR activation secondary to cigarette smoke exposure to skeletal muscle bioenergetic deficits in male, but not female, mice. AHR activation is a likely contributor to the decline in muscle oxidative capacity observed in smokers and AHR antagonism may provide a therapeutic avenue aimed to improve muscle function in COPD.

## Introduction

Tobacco smoking is a leading cause of death and preventable disease, including chronic obstructive pulmonary disease (COPD). Patients with COPD, as well as smokers without COPD, display symptoms of limb muscle dysfunction including atrophy, weakness and exercise intolerance, fibre type shifts, and reduced muscle oxidative capacity.^1^, ^2^, ^3^, ^4^, ^5^, ^6^, ^7^, ^8^ Preclinical studies involving tobacco smoke exposure yield similar muscle affect.^9^, ^10^, ^11^ While progress has been made toward understanding the impact of limb muscle dysfunction on COPD patient outcomes, the mechanisms that contribute to intrinsic muscle dysfunction with tobacco smoking are ill‐defined.

Several constituents of tobacco smoke activate the aryl hydrocarbon receptor (AHR), a ligand‐activated transcription factor which upregulates xenobiotic enzymes. Although transient AHR activation is necessary to degrade toxins, chronic AHR activity can lead to pathology,^12^ and some reports have suggested mitochondrial mechanisms may be involved.^13^, ^14^, ^15^ Recent work has revealed that cigarette smoke increases AHR signalling in both mice and humans, and that AHR antagonism can attenuate myotube atrophy.^11^ However, the mechanisms linking constitutive AHR activity to muscle pathology are incompletely understood. On this basis, the goal of this study was to determine the role of chronic cigarette smoke‐induced AHR activation in skeletal muscle pathophysiology. Experiments involving chronic cigarette smoke exposures were conducted using a newly developed muscle‐specific conditional AHR knockout mouse. Additional experiments employing viral‐mediated ectopic expression of a mutant AHR transgene developed to produce chronic activation of the AHR without cigarette smoking were performed. It was hypothesized that muscle‐specific knockout of the AHR would attenuate muscle atrophy, weakness, and biochemical deficiencies limiting oxidative capacity of the muscle caused by chronic cigarette smoke exposure, whereas viral‐mediated chronic AHR activation in muscle would cause muscle atrophy, weakness, and mitochondrial dysfunction.

## Methods

### Animals

We generated muscle‐specific AHR knockout mice (AHR^mKO^) by breeding conditionally floxed AHR mice (AHR^tm3.1Bra^/J, Jackson Laboratories, Stock No. 006203) with HSA‐MCM mice that express MerCreMer double fusion protein under the control of the human ACTA1 promoter (Tg (ACTA1‐cre/Esr1*)2Kesr/J, Jackson Laboratories, Stock No. 025750). Muscle‐specific AHR knockout was induced by intraperitoneal injection of tamoxifen (80 mg/kg) for five consecutive days. Muscle‐specific DNA recombination was confirmed using PCR amplification of genomic DNA using primers flanking Exon 2 (Forward = 5‐atcttgtgtcaggaacaggccatc‐3′ and Reverse = 5′‐ggtacaagtgcacatgcctgc‐3′). All other experiments involved C57BL6J mice (*n* = 80) obtained from Jackson Laboratories (Stock No. 000664). All animal experiments adhered to the Guide for the Care and Use of Laboratory Animals from the Institute for Laboratory Animal Research, National Research Council, Washington, D.C., National Academy Press, 2011, and any updates. All procedures were approved by the Institutional Animal Care and Use Committee of the University of Florida (Protocol 202009766).

### Cigarette smoke exposure

Three weeks following the last intraperitoneal injection of tamoxifen, AHR^mko^ mice were exposed to either room air or tobacco smoke (TS) for 16 weeks with exposures lasting 2 h per day, 5 days per week. The concentration of TS was measured during each smoke exposure using a standard flow meter and a filter paper per manufacturer protocol. The average TS concentration across the 16 weeks was 220 ± 80 mg/m^3^. Mice in the air‐exposed group were kept in the same barrier facility as the TS‐exposed mice, but not exposed to TS.

### Adeno‐associated virus construction and delivery

To accomplish muscle cell‐specific overexpression of transgenes, the human skeletal actin (ACTA1; termed HSA herein) promoter was PCR amplified from human DNA isolated from a donor muscle biopsy. The AAV‐HSA‐GFP plasmid was developed by inserting the HSA promoter and GFP (ZsGreen1) into the promoterless AAV vector (Cell BioLabs, Cat. No. VPK‐411‐DJ) using In‐Fusion Cloning (Takara Bio, Cat. No. 638911). Similarly, the mouse AHR, including ligand binding domain, was PCR amplified from cDNA obtained from a C57BL6J mouse and inserted downstream of the HSA promoter. To generate a constitutively active AHR (CAAHR) vector, the mouse AHR coding sequence was PCR amplified from cDNA obtained from a C57BL6J mouse such that the ligand binding domain (amino acids 277–418) was deleted. The resulting plasmids were packaged using AAV2/9 serotype by Vector Biolabs (Malvern, PA). AAV9 was delivered via intramuscular injections of the gastrocnemius, tibialis anterior (TA), extensor digitorum longus (EDL) muscles of both legs at a dosage of 5E+11 vg/limb.

### Preparation of permeabilized myofibre bundles

Mice were anaesthetized by intraperitoneal injection of ketamine (90 mg/kg) and xylazine (10 mg/kg) and the gastrocnemii removed, blotted to remove excess blood, and weighed. The red portions of the gastrocnemii were then carefully dissected using a pair of sharp scissors and then placed into pre‐cooled buffer A (CaK_2_EGTA (2.77 mM), K_2_EGTA (7.23 mM), MgCl_2_ (6.56 mM), dithiothreitol (0.5 mM), K‐MES (50 mM), imidazole (20 mM), taurine (20 mM), Na_2_ATP (5.3 mM), phosphocreatine (15 mM), pH 7.3 at 4°C). The remaining portions of the gastrocnemii were either used to isolate skeletal muscle mitochondria (see below) or snap‐frozen in liquid nitrogen and stored at −80°C for subsequent analysis. Thin fibre bundles from the red gastrocnemii were carefully separated along their fibre orientation in buffer A at 4°C. Myofibre bundles were then permeabilized by mild shaking for 30 min in buffer A supplemented with saponin (50 μg/mL). After permeabilization, fibre bundles were immediately washed for 10 min in buffer D (MgCl_2_‐6H_2_O (5 mM), K‐MES (105 mM), KCl (30 mM), KH_2_PO_4_ (10 mM), EGTA (1 mM), BSA (2.5 g/L), pH 7.2 at room temperature), then blotted dry to remove excess water and weighed prior to measurement of oxygen consumption or H_2_O_2_ emission.

### Isolation of skeletal muscle mitochondria

To isolate skeletal muscle mitochondria, the gastrocnemius muscle was rapidly dissected and placed in ice‐cold PBS supplemented with 10 mM EDTA. The muscle was carefully trimmed of fat and connect tissues, minced on ice, and digested for 5‐min with 0.025% *w*/*v* trypsin (Millipore‐Sigma, Cat. No. T4799). Following trypsin digestion, the tissue was centrifuged for 5 min at 200× *g* and the supernatant was aspirated. The digested tissue pellet was resuspended in Buffer C (MOPS (50 mM), KCl (100 mM), EGTA (1 mM), MgSO_4_ (5 mM), bovine serum albumin (BSA; 2 g/L); pH = 7.1) and then homogenized via a glass‐Teflon homogenizer (Wheaton) and subsequently centrifuged at 800× *g* for 10 min. The tissue pellet was discarded, and the resulting supernatant was centrifuged at 10 000× *g* for 10 min to pellet mitochondria. All steps were performed at 4°C. The mitochondrial pellet was gently washed to remove any damaged mitochondria and then re‐suspended in Buffer B [MOPS (50 mM), KCl (100 mM), EGTA (1 mM), MgSO_4_ (5 mM); pH = 7.1] and protein concentration was determined using bicinchoninic acid protein assay (ThermoFisher Scientific, Cat. No. A53225).

### Assessment of mitochondrial oxygen consumption and hydrogen peroxide emission

For experiments involving isolated mitochondria, respiratory function was assessed at 37°C in buffer D (in mmol/L) supplemented with creatine monohydrate (5 mM), using the OROBOROS O2K Oxygraph. H_2_O_2_ production was assessed using the Amplex Ultra Red/horseradish peroxidase detection system. Detailed methods for mitochondrial function assessments can be found in the supporting information.

### Nerve‐mediated muscle contractile function

Skeletal muscle contractile function was assessed in the EDL muscle *in situ* using stimulation of the peroneal nerve. Mice were anaesthetised with ketamine (90 mg/kg) and xylazine (10 mg/kg) and the distal EDL tendon was carefully isolated, and a silk ligature was tied and attached to the lever arm of the force transducer (Cambridge Technology; Model: 2250). Force frequency curves were created by stimulating at 1, 25, 50, 75, 100, 125, 150, and 175 Hz with 1‐min rest between contractions. Muscle fatiguability was assessed by delivering 50 Hz contractions every 2 s for 3 min. Recovery from fatigue was examined by delivering additional 50 Hz contractions at 1, 3, 5, and 10‐min post‐fatigue testing. Specific force was calculated by normalizing forces to the muscle weight.

### Skeletal muscle histology and immunofluorescence microscopy

10‐μm‐thick transverse sections of the TA, EDL, and Soleus (Sol) muscles were cut using a cryotome (Leica CM3050S) and collected on slides. Skeletal myofibre cross‐sectional area (CSA) was assessed by staining sections with 5 μg/mL wheat germ agglutinin conjugated to AlexaFluor‐647 (Invitrogen, Cat. No. W32466), washed with PBS, and cover slipped with Vectashield Hardmount with DAPI (Vector Laboratories, Cat. No. H‐1500). Images were obtained at 20× magnification using an Evos FL2 Auto microscope (ThermoFisher Scientific) and tiled images of the entire muscle section were analysed using MuscleJ.^16^

### RNA‐isolation and qRT‐PCR

Total RNA was extracted from gastrocnemius muscle using a Direct‐zol RNA MiniPrep kit (Zymo Research, R2052) following the manufacturer's direction. cDNA was generated from 500 ng of RNA using the LunaScript RT Supermix kit (New England Biolabs, E3010L) according to the manufacturer's directions. Real‐time PCR (RT‐PCR) was performed on a Quantstudio 3 (ThermoFisher Scientific) using Luna Universal qPCR master mix (New England Biolabs, M3003X) and the following primers: *Ahr* (Forward‐AACATCACCTATGCCAGCCG, Reverse‐GGTCTCTGTGTCGCTTAGAAGG), *Cyp1a1* (Forward‐CAGCCTTCCCAAATGGTTTA, Reverse‐GCCTGGGCTACACAAGACTC), and *L32* (Forward‐TTCCTGGTCCACAATGTCAA, Reverse‐GGCTTTTCGGTTCTTAGAGGA) was used as the housekeeping control. Relative gene expression was calculated using 2^−ΔΔCT^ from the relevant control group.

### Analysis of mitochondrial content

Skeletal muscle mitochondrial content was analysed using immunoblotting with an antibody cocktail targeting protein subunit of each electron transport system complex (Abcam, Cat. No. ab110413; 1:1000 dilution), as well as citrate synthase activity via a commercial kit (Millipore‐Sigma, Cat. No. CS0720).

### Gene expression profiling in human muscle specimens

To examine the AHR signalling pathway in human muscle specimens from patients with chronic obstructive pulmonary disease (COPD) and age‐matched healthy controls that were non‐smokers, we analysed a publicly available microarray dataset (GEO100281).^17^ Gene expression data were RMA‐treated using Affymetrix Power Tools (APT, 1.16.1) and imported into R (version 4.2.0).

### RNA sequencing in mouse muscle specimens

Total RNA was extracted from gastrocnemius muscle using the Direct‐zol RNA MiniPrep kit (Zymo Research, Cat. No. R2052). Library preparation and mRNA sequencing via PolyA selection was performed by Genewiz (Azenta Life Science, South Plainfield, NJ). Paired‐end 150 bp reads were sequenced on an Illumina HiSeq 4,000. Detailed description of data processing and analysis can be found in the supporting information. The raw data have been deposited in NCBI's Gene Expression Omnibus under accession numbers GSE225607 and GSE225670.

### Statistical analysis

Data are presented as mean ± SD. Normality of data was tested with the Shapiro–Wilk test and inspection of QQ plots. Comparisons between two groups were performed by Student's unpaired two‐tailed *t*‐test. If variances were found to be different between groups, an unpaired *t*‐test with Welch's correction was performed. Comparisons of data with more than two groups were performed using two‐way ANOVA with Šidák's post‐hoc testing for multiple comparisons when significant interactions were detected. All statistical analysis was performed in GraphPad Prism (Version 9.0). *P* < 0.05 was considered statistically significant.

## Results

### The aryl hydrocarbon receptor pathway is activated in human chronic obstructive pulmonary disease muscle and changes temporally following acute cigarette smoke exposure in mouse muscle

**Figure 1:**
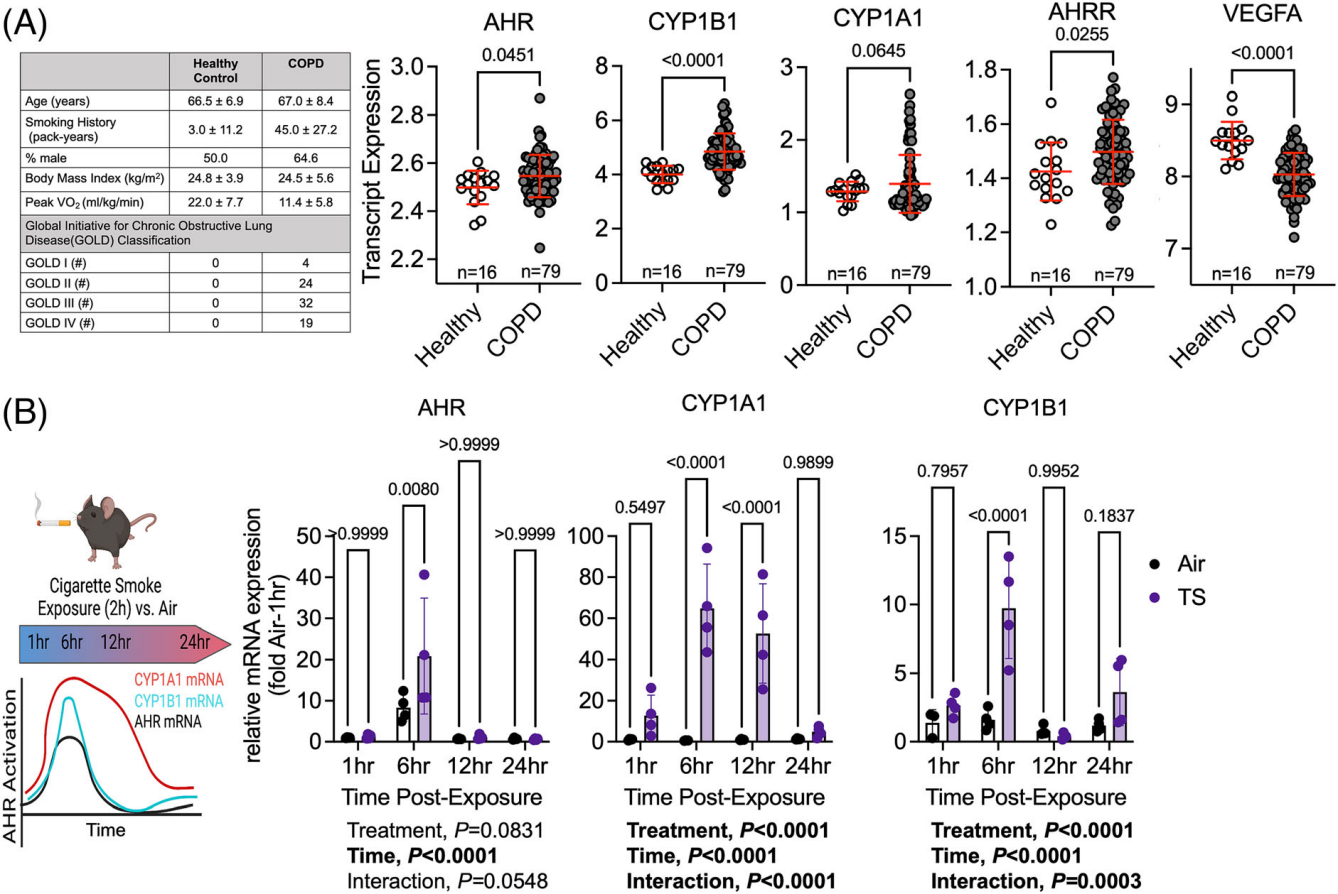
The AHR is activated by tobacco/cigarette smoke in human and murine muscles. (A) Physical characteristics of participants from which human muscle specimens were derived from patients with chronic obstructive pulmonary disease (COPD) and age‐matched controls. Data were obtained from a publicly available microarray dataset (GEO100281) and normalized mRNA expression of select AHR signalling pathway genes are shown. (B) mRNA analysis in skeletal muscle obtained from male C57BL6J mice subjected to either air control or an acute (2 h) cigarette smoke exposure (*n* = 4/group). Muscles were harvested across a 24 h period after the exposure to assess the temporal nature of AHR activation. Analysis in panel (A) involved unpaired Student's *t*‐test with FDR correction for multiple comparisons, whereas panel (B) involved a two‐way ANOVA Šidák's post‐hoc testing for multiple comparisons when appropriate. Error bars represent the standard deviation.

We analysed a publicly available microarray dataset developed from adult controls that were non‐smokers and patients with COPD^17^ for alterations in the AHR pathway. Similar to the mRNA analysis in human skeletal muscle from active smokers,^11^ signficant elevations in the mRNA levels of the *AHR*, *CYP1B1*, *AHRR*, as well as a trending increase in *CYP1A1* (*P* = 0.0645) and decrease in *VEGFA* (Figure 1A) provide evidence of AHR activation in skeletal muscle of patients with COPD.

Next, we examined the temporal nature of AHR activation following a single exposure to cigarette smoke. C57BL6 mice were subjected to a 2‐h cigarette smoke exposure (average total suspended particulates = 235 mg/m^3^) or air (control group). Gastrocnemius muscle was harvested 1, 6, 12, and 24 h following exposure and mRNA levels of key AHR pathway genes were assessed (Figure 1B). Significant treament effects were found for both *Cyp1a1* and *Cyp1b1* (*P* < 0.0001 for both) indicating that cigarette smoke exposure acutely activates the AHR pathway. Thus, it is reasonable to conclude that the typical human smoker with multiple exposures across the waking day experiences chronic AHR activation in muscle.

### Muscle‐specific deletion of the aryl hydrocarbon receptor has no impact on muscle mass or myofibre area in mice exposed to chronic cigarette smoke

**Figure 2:**
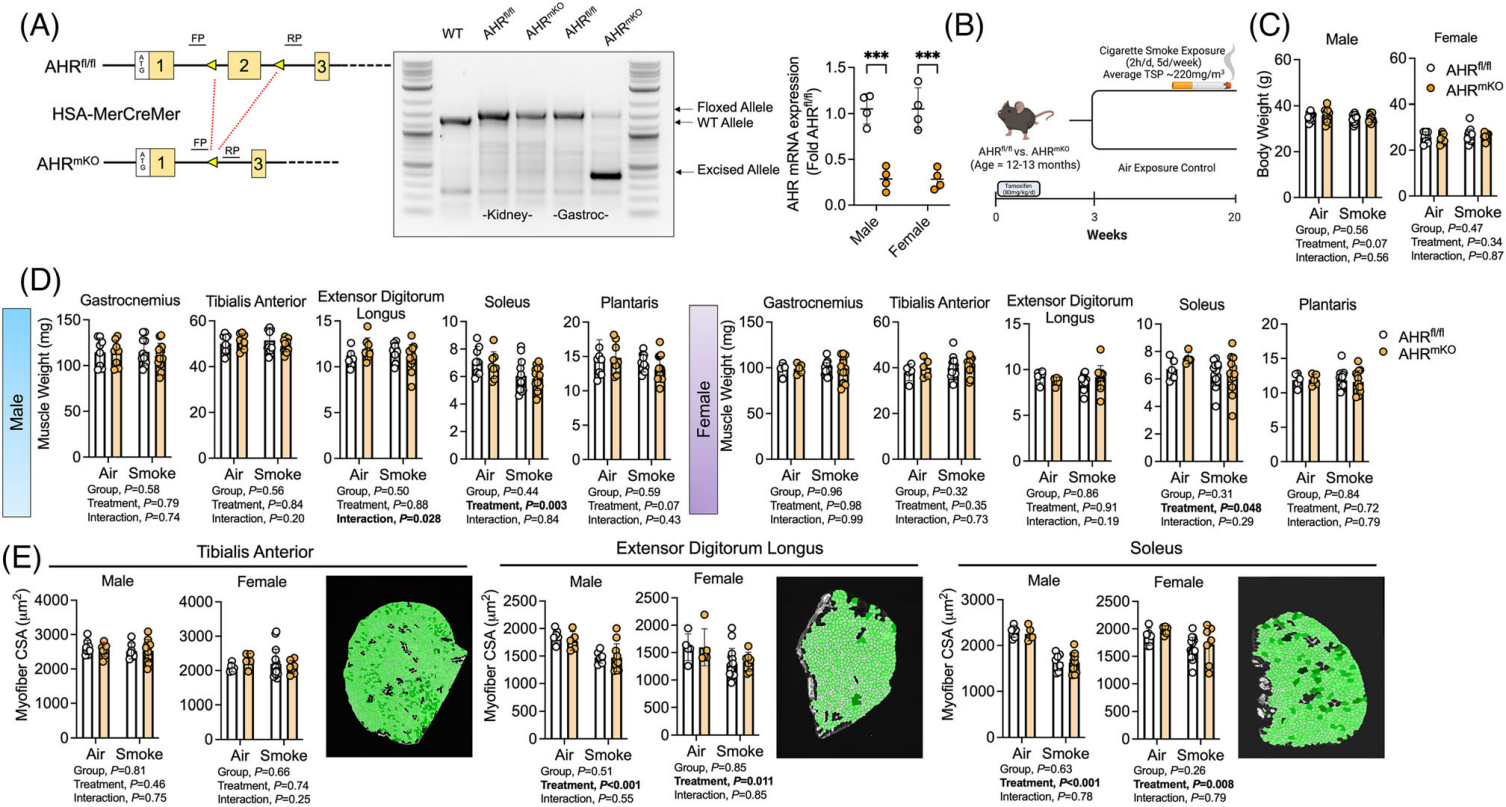
Muscle‐specific deletion of the AHR has no impact on muscle mass or myofibre area in mice exposed to chronic cigarette smoke. (A) Graphical depiction of the generation of a muscle‐specific conditional AHR knockout mouse (AHR^mKO^) and primers used to confirm cell‐specific DNA recomination. Evidence of muscle‐specific DNA recombination in AHR^mKO^ mice treated with tamoxifen and qPCR for AHR mRNA expression (*n* = 4/group/sex). (B) Graphical depiction of the chronic cigarette smoking protocol. (C) Body weight of mice (*n* = 8–14/group/sex). (D) Muscle wet weights obtained for hindlimb muscles of AHR^fl/fl^ and AHR^mKO^ mice (*n* = 5–14/group/sex). (E) Myofibre cross section area (CSA) of hindlimb muscles from mice (*n* = 5–14/group/sex). Analysis involved a two‐way ANOVA Šidák's post‐hoc testing for multiple comparisons when appropriate. Error bars represent the standard deviation.

To explore the role of chronic AHR activation in myofibres with chronic repeated cigarette smoke exposures, we generated a conditional, muscle‐specific AHR knockout mouse (AHR^mKO^) (Figure 2A). Delivery of tamoxifen to drive expression of the muscle‐specific Cre recombinase resulted in the expected DNA editing only in skeletal muscle as shown by traditional PCR and real‐time quantitative PCR (Figure 2A). AHR^mKO^ and littermates lacking Cre (AHR^fl/fl^) were subjected to chronic repeated (5 days per week for 16 weeks) cigarette smoke exposures (Smoke) or air control (Air) beginning 3 weeks after tamoxifen delivery (Figure 2B). Cigarette smoke exposures averaged a total suspended particulates (TSP) of 220 ± 80 mg/m^3^ across the intervention. Following the intervention, neither body weight (Figure 2C) nor hindlimb muscle weights were different between control (AHR^fl/fl^) and AHR^mKO^ mice (Figure 2D), indicating that muscle‐specific AHR ablation does not impact muscle mass in mice with chronic cigarette smoke exposure. There was no significant treatment effect in most muscle weights indicating that chronic cigarette smoke exposure did not produce atrophy in these aged mice. The only exception was that chronic cigarette smoke exposure decreased the soleus muscle weight (*P* = 0.003 and *P* = 0.048 for male and female, respectively), suggesting a potential fibre type specific effect may have been present. Myofibre cross‐sectional areas (CSA) of the tibialis anterior, extensor digitorum longus, and solues muscles were also not different between AHR^fl/fl^ and AHR^mKO^ mice (all group effects *P* > 0.26; Figure 2E).

### Muscle‐specific deletion of the aryl hydrocarbon receptor has no impact on muscle strength or fatiguability

**Figure 3:**
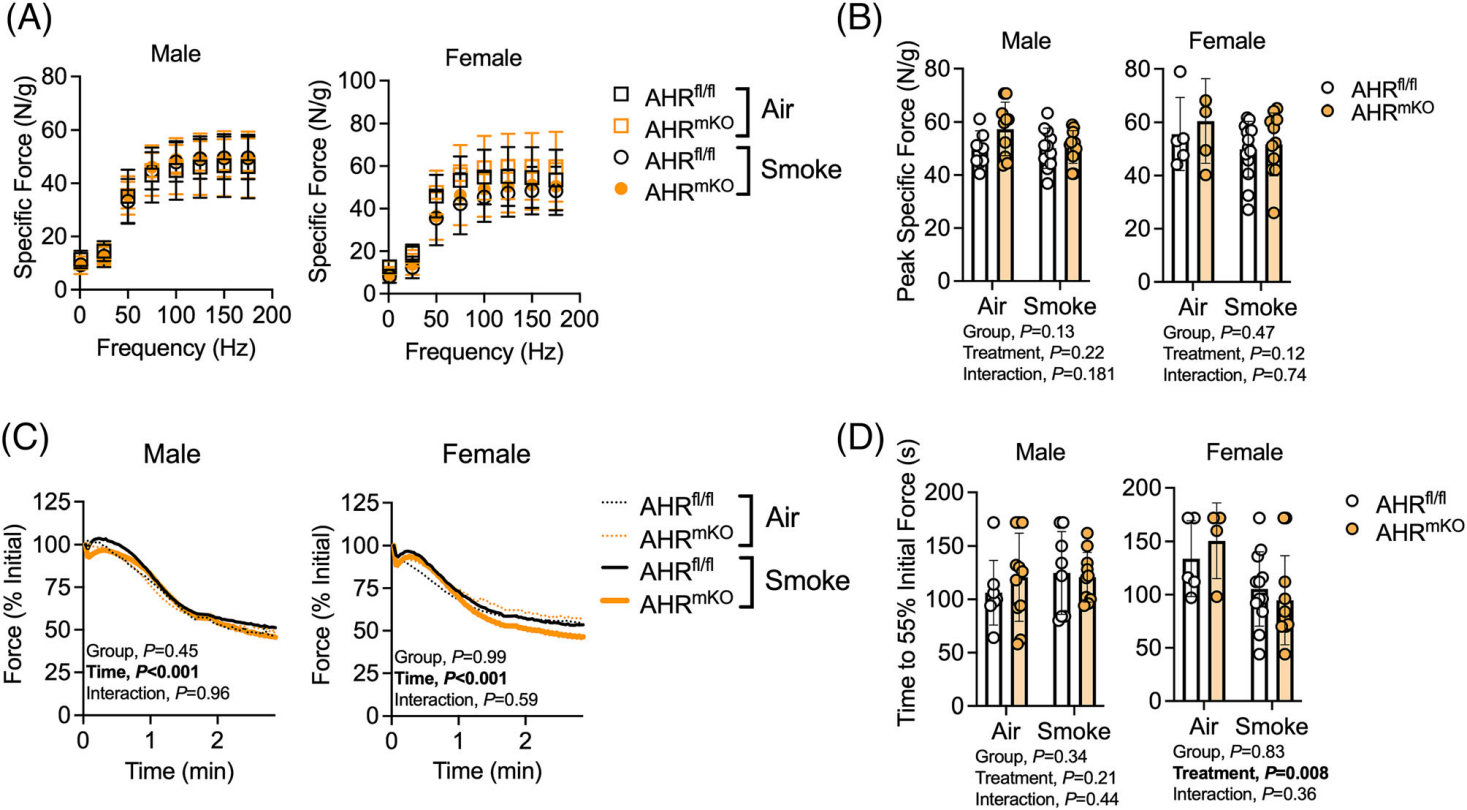
Muscle‐specific deletion of the AHR has no impact on isometric force or fatigue levels in mice exposed to chronic cigarette smoke. (A) Force‐frequency curves of the extensor digitorum longus muscle performed using nerve‐mediated contraction in male and female mice (*n* = 5–14/group/sex). (B) Quantification of the peak specific isometric force levels from panel (A). (C) Isometric fatigue testing results (force presented at a percentage of initial) in male and female mice. (D) Quantification of the time it took to reach 55% of the initial force (*n* = 5–14/group/sex). Analysis involved a two‐way ANOVA Šidák's post‐hoc testing for multiple comparisons when appropriate. Error bars represent the standard deviation.

Next, we performed measures of muscle function using nerve‐mediated stimulation. Force‐frequency curves in AHR^fl/fl^ and AHR^mKO^ mice are shown in Figure 3A, and indicate that deletion of the AHR in skeletal muscle did not impact specific force levels in mice exposed to chronic cigarette smoke or air controls. Quantification of the peak specific force confirmed this (Figure 3B). AHR deletion did not impact muscle fatigue in mice chronically exposed to cigarette smoke (Figure 3C). Quantitative comparison of the time required to reach 55% of the initial force confirmed the absence of a group effect, but uncovered a significant treatment effect only in female mice (*P* = 0.008), indicating that smoking caused more rapid fatigue in female mice (Figure 3D).

### Muscle‐specific deletion of aryl hydrocarbon receptor increases mitochondrial function in male mice exposed to chronic cigarette smoke

**Figure 4:**
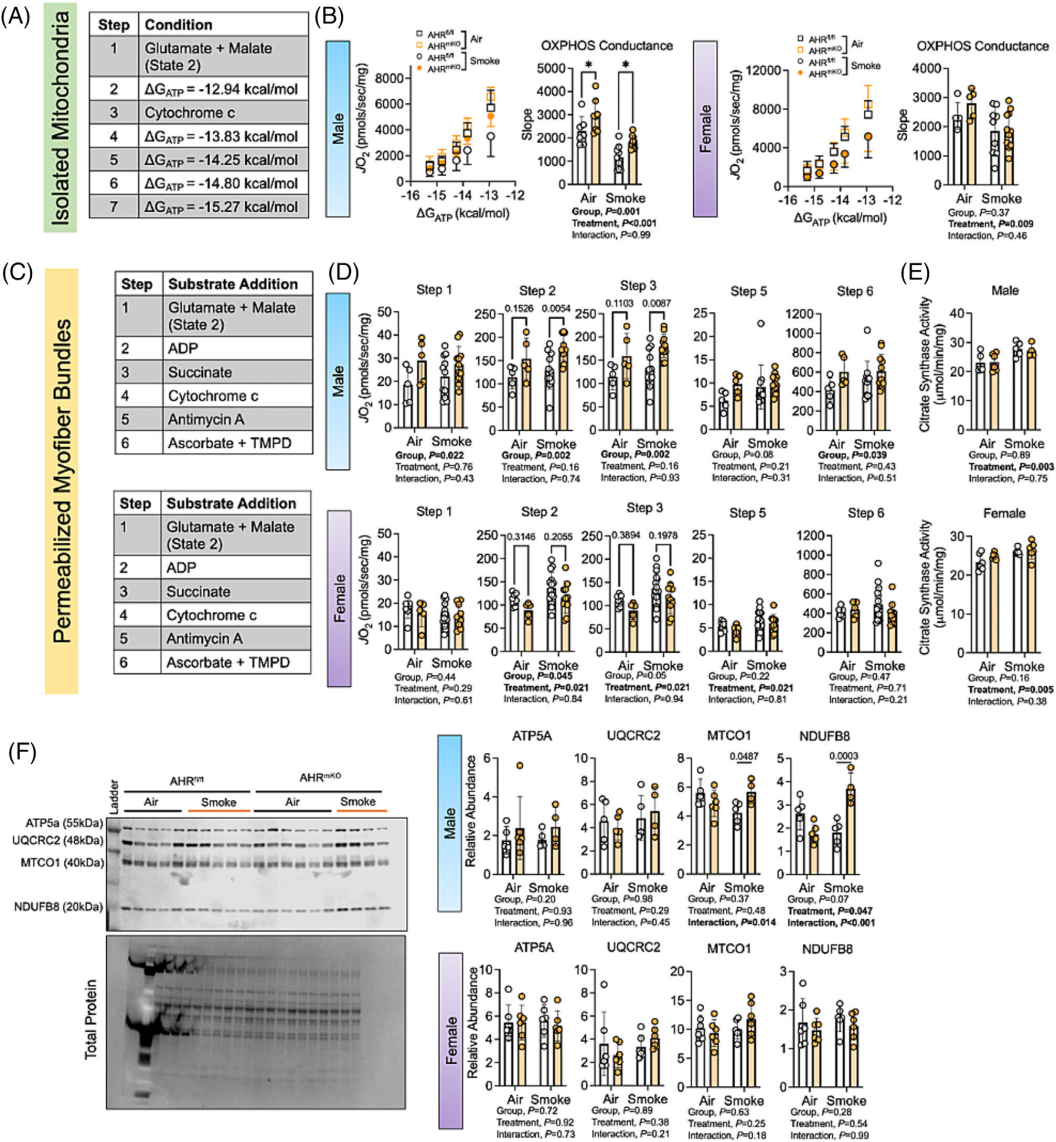
Muscle‐specific deletion of AHR increases mitochondrial respiratory function in male mice exposed to chronic cigarette smoke. (A) A description of the substrate protocol for isolated muscle mitochondrial analysis. (B) Mitochondrial respiratory flux (*J*O_2_) in isolated mitochondrial from skeletal muscle (gastrocnemius) in male and female mice, as well as quantification of OXPHOS conduction, representing the slope in the *J*O_2_ versus ΔG_ATP_ graph (*n* = 4–11/group/sex). (C) Respiratory protocol performed in permeabilized myofibre bundles prepared from the red gastrocnemius muscle of mice. (D) Respiration rates in permeabilized myofibre experiments (*n* = 5–14/group/sex). (E) Citrate synthase activity assay results measured in gastrocnemius muscle lysate (*n* = 4–6/group/sex). (F) Immunoblotting analysis of OXPHOS complex subunits in lysate preapred from the gastrocnemius muscle (*n* = 4–6/group/sex). Analysis in all panels was done using two‐way ANOVA Šidák's post‐hoc testing for multiple comparisons when appropriate. Error bars represent the standard deviation.

Previous reports have shown that chronic cigarette smoking can negatively impact skeletal muscle oxidative capacity in both humans and rodents.^1^, ^2^, ^11^, ^18^ Thus, we explored skeletal muscle mitochondrial respiratory function using two complimentary approaches involving measures performed in isolated mitochondria and in permeabilized myofibre bundles. In isolated mitochondria, we employed a creatine kinase clamp protocol to study mitochondrial oxidative phosphorylation (OXPHOS) under physiologically relevant levels of energy demand^19^ (Figure 4A). Chronic cigarette smoke exposure resulted in a signficant treatment effect in both male (*P* < 0.001) and female (*P* = 0.009) mice, demonstrating an impairment in mitochondrial OXPHOS occurred in the Smoke groups (Figure 4B). OXPHOS conductance was significantly higher in male AHR^mKO^ mice compared to AHR^fl/fl^ in both air control and Smoke groups (Figure 4B). Interestingly, this effect was not observed in female mice. Using permeabilized myofibre bundles (Figure 4C), a treatment effect of cigarette smoking was not observed in male mice, but was found in some substrate conditions (ADP stimulated) for female mice (Figure 4D). Male AHR^mKO^ mice exposed to chronic cigarette smoke had higher bundle respiration rates compared to AHR^fl/fl^ mice under state 2 (no ADP), state 3 (ADP‐stimulated), and complex IV‐dependent respiratory conditions (Figure 4D). To explore the mechanisms underlying the discrepant finding between isolated mitochondrial and permeabilized myofibre bundles, we performed assessments of mitochondrial content in the gastrocnemius muscle. Citrate synthase activity, a biomarker of mitochondrial content in muscle, was significantly higher in smoke exposed male (treatment effect, *P* = 0.003) and female (treatment effect, *P* = 0.005) mice, but no effect of AHR deletion was observed (Figure 4E). Immunoblotting for OXPHOS protein complex subunits also revealed significant treatment and interaction effects in male mice for MTCO1 and NDUFB8 (Figure 4F); however, these effects were not detect in female mice. Taken together, these findings indicate that cigarette smoke induced defects in OXPHOS (i.e., energetic stress) stimulate a compensatory increase in mitochondrial biogenesis. Analyses of mitochondrial hydrogen peroxide emission/production indicated that hydrogen peroxide production in isolated mitochondria was not affected by cigarette smoke exposure or the deletion of the AHR under energized conditions (Figure S1A,B). In permeabilized myofibre bundles (Figure S1D), mitochondrial hydrogen peroxide emission was significantly increased in Smoke exposed mice when mitochondria were energized but in the absence of ADP in both males (*P* = 0.041) and females (*P* = 0.002). Following the addition of 2 mM ADP, this increased mitochondrial hydrogen peroxide emission was only retained in female mice (*P* = 0.048). Regardless of sample preparation method or substrate conditions, the deletion of the AHR had no impact on mitochondrial hydrogen peroxide emission/production.

### Muscle‐specific expression of a constitutively active aryl hydrocarbon receptor in healthy mice does not impact muscle size

**Figure 5:**
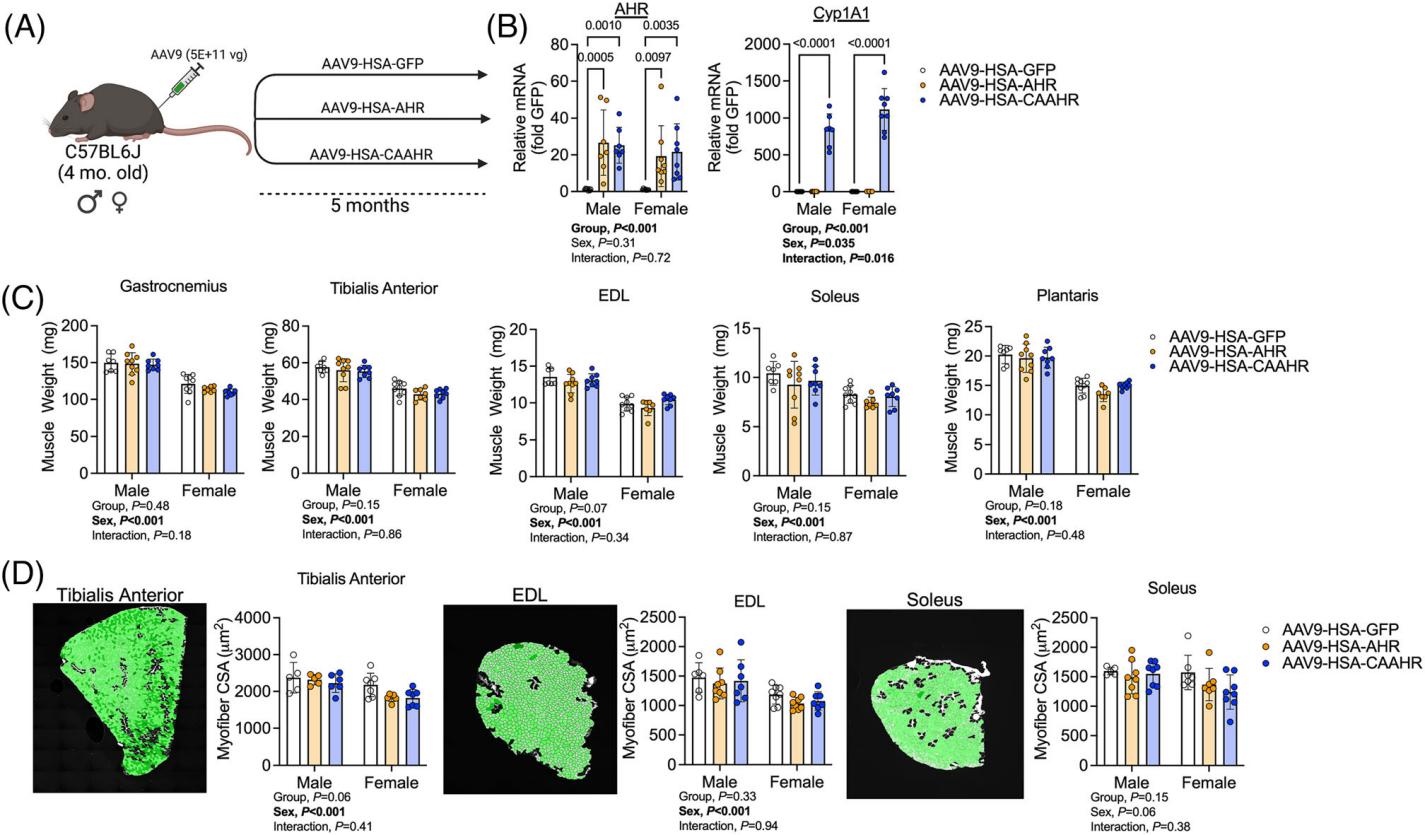
Muscle‐specific expression of a constitutively active AHR (CAAHR) in healthy mice does not impact muscle size. (A) Graphical depiction of the experimental approach to deliver muscle‐specific AAV's driven by the human skeletal actin (HSA, ACTA1) promoter. (B) qPCR validation of both AHR overexpression and constitutive AHR activation (CAAHR) in skeletal muscle from male and female mice (*n* = 7–8/group/sex). (C) Muscle wet weights obtained for hindlimb muscles (*n* = 7–9/group/sex). (D) Myofibre cross section area (CSA) of hindlimb muscles from mice (*n* = 5–7/group/sex). Analysis involved a two‐way ANOVA Šidák's post‐hoc testing for multiple comparisons when appropriate. Error bars represent the standard deviation.

Next, we sought to examine the specific role of AHR activation in skeletal muscle under conditions without chronic cigarette smoke exposure using an adeno‐associated virus (AAV) approach to ectopically express either the full mouse AHR coding sequence or a mutated AHR sequence that was constitutively active (CAAHR) driven by a muscle‐specific promoter (Figure 5A). As expected, *Ahr* expression was significantly increased in both AAV9‐HSA‐AHR and AAV9‐HSA‐CAAHR treated male and female mice compared with AAV9‐HSA‐GFP (Figure 5B). However, *Cyp1a1* levels were increased only in AAV9‐HSA‐CAAHR muscles (Figure 5B). Hindlimb muscle weights (Figure 5C) and myofibre CSA (Figure 5D) were unaffected by ectopic expression of CAAHR or AHR.

### Muscle‐specific expression of a constitutively active aryl hydrocarbon receptor exacerbates muscle fatigue

**Figure 6:**
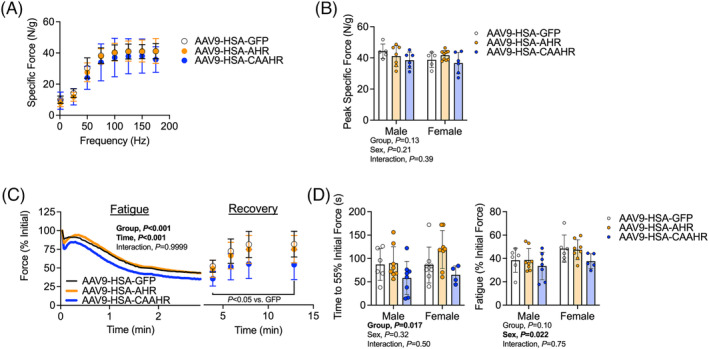
Muscle‐specific expression of a constitutively active AHR (CAAHR) has no impact on isometric force but exacerbates fatigue in mice. (A*)* Force‐frequency curves of the extensor digitorum longus muscle performed using nerve‐mediated contraction in male and female mice (*n* = 5–9/group/sex). (B) Quantification of the peak specific isometric force levels from panel (A). (C) Isometric fatigue testing results (force presented at a percentage of initial) in male and female mice (*n* = 4–8/group/sex). (D) Quantification of the time it took to reach 55% of the initial force, and the % fatigue at the end of the protocol in male and female mice (*n* = 4–8/group/sex). Analysis involved a two‐way ANOVA Šidák's post‐hoc testing for multiple comparisons when appropriate. Error bars represent the standard deviation.

AAV‐mediated expression of the full AHR or CAAHR transgenes did not impact submaximal or peak specific force (Figure 6A,B). However, a significant group effect was observed in the rate of muscle fatigue development as well as delayed force recovery after fatigue in AAV9‐HSA‐CAAHR treated mice (Figure 6C). AAV9‐HSA‐CAAHR muscles exhibited a shorter time to reach 55% of their initial force (*P* = 0.017), confirming a faster rate of fatigue.

### Muscle‐specific expression of a constitutively active aryl hydrocarbon receptor impairs mitochondrial OXPHOS

**Figure 7:**
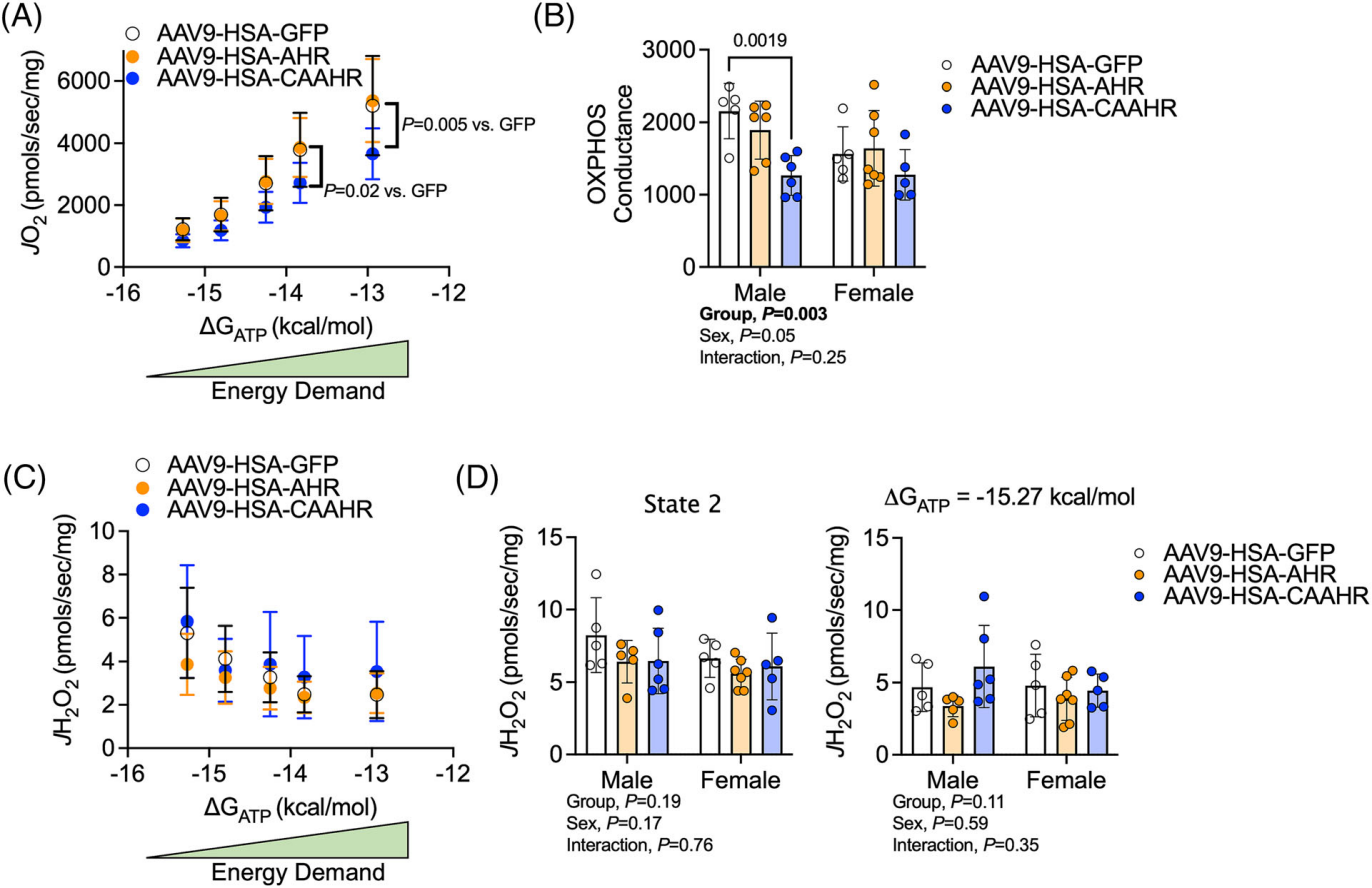
Muscle‐specific expression of a constitutively active AHR (CAAHR) impairs mitochondrial OXPHOS in mice. (A) Mitochondrial respiratory flux (*J*O_2_) in isolated mitochondrial from skeletal muscle (gastrocnemius) in male and female mice across physiologically relevant levels of energy demand (ΔG_ATP_) (*n* = 5–7/group/sex). (B) Quantification of OXPHOS conduction, representing the slope in the *J*O_2_ vs. ΔG_ATP_ graph (*n* = 5–7/group/sex). (C) Mitochondrial hydrogen peroxide emission (*J*H_2_O_2_) in male and female mice across physiologically relevant levels of energy demand (ΔG_ATP_) (*n* = 5–7/group/sex). (D) Quantification of *J*H_2_O_2_ under State 2 (no energy demand) and conditions similar to resting energy demand (*n* = 5–7/group/sex). Analysis involved a two‐way ANOVA Šidák's post‐hoc testing for multiple comparisons when appropriate. Error bars represent the standard deviation.

To explore if expression of CAAHR could recapitulate the mitochondrial OXPHOS deficit detected in mice with chronic cigarette smoke exposure, we isolated mitochondria from the entire gastrocnemius muscle. This approach was preferred over the permeabilized bundle preparation because potential heterogeneity in AAV infection amongst the total muscle fibre pool that could adversely affect our ability to detect a change in small muscle bundles versus isolating mitochondria from the entire muscle. Mitochondrial respiration was significantly lower in AAV9‐HSA‐CAAHR treatment mice at the two highest levels of energy demand (Figure 7A). Quantification of the OXPHOS conductance identified a significant group effect (*P* = 0.003) indicating that AAV9‐HSA‐CAAHR treated mice had lower OXPHOS conductance (Figure 7B). Notably, there was a trend for sex differences (*P* = 0.05), and the magnitude of impairment in OXPHOS conductance was larger in male mice compared to female mice infected with AAV9‐HSA‐CAAHR. CAAHR had no impact on mitochondrial H_2_O_2_ production (Figure 7C,D).

### Aryl hydrocarbon receptor‐mediated transcriptional activity alterations the mitochondrial transcriptome

**Figure 8:**
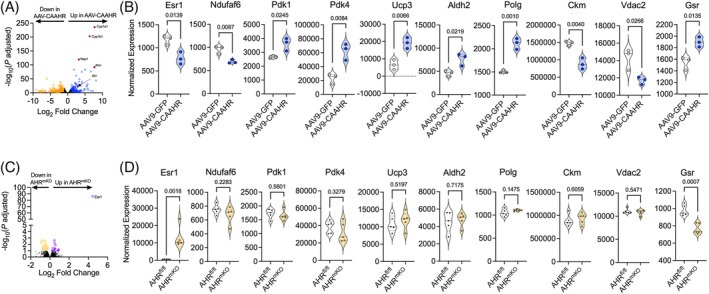
RNA sequencing results identify AHR‐responsive gene expression changes in muscle. (A) Volcano plot showing differentially expressed genes in AAV9‐HSA‐CAAHR and AAV9‐HSA‐GFP muscles from male mice (*n* = 3/group). (B) Violin plots of select differentially expressed genes in AAV9‐HSA‐CAAHR and AAV9‐HSA‐GFP muscles from male mice. (C) Volcano plot showing differentially expressed genes in AHR^fl/fl^ and AHR^mKO^ muscle harvested 72 h post‐smoke from male mice (*n* = 5/group). (D) Violin plots of select differentially expressed genes in in AHR^fl/fl^ and AHR^mKO^ muscle. Analysis involved a two‐tailed unpaired *t*‐test with false discovery rate adjustment.

Next, we performed RNA sequencing analyses on muscles from AAV9‐HSA‐GFP and AAV9‐HSA‐CAAHR treated male mice. A volcano plot of differential RNA expression is shown in Figure 8A. AAV9‐HSA‐CAAHR muscles displayed significantly higher expression of AHR pathway genes including *Ahr*, *Ahrr*, *Cyp1a1*, *Cyp1b1*, and *Nqo1*. Violin plots of differentially expressed genes shows decreased expression of *Esr1*, *Ndufaf6*, and *Vdac2* in AAV9‐HSA‐CAAHR muscles (Figure 8B). AAV9‐HSA‐CAAHR muscles also displayed upregulated expression of several negative regulators of mitochondrial energetics including *Pdk1*, *Pdk4*, *Ucp3*, as well as the mtDNA repair enzyme *Polg* and a reactive aldehyde dehydrogenase *Aldh2* (Figure 8B). To confirm the AHR responsiveness of these genes, we also performed RNA sequencing analysis on muscles from AHR^fl/fl^ and AHR^mKO^ male mice that underwent cigarette smoke exposure. However, muscles from AHR^fl/fl^ and AHR^mKO^ mice were harvested 48–72 h following the final cigarette smoke exposure, a time when AHR activation returns to baseline levels (Figure 1B). A volcano plot of RNA expression in AHR^mKO^ muscle is shown in Figure 8C. In AHR^mKO^ muscle, examination of the mitochondrial‐associated genes that were impacted by CAAHR expression demonstrated that deletion of the AHR abolished these effects (Figure 8D).

## Discussion

The present study examined the role of chronic AHR activation in the skeletal myopathy associated with chronic cigarette smoke exposure. Chronic cigarette smoke exposure was found to significantly impair skeletal muscle mitochondrial energetics in both male and female mice. Deletion of the AHR in skeletal muscle significantly improved mitochondrial OXPHOS and respiratory capacity in male mice, but not their female counterparts exposed to cigarette smoke. Conversely, expression of a CAAHR mutant in myofibres of healthy mice significantly impaired mitochondrial energetics and exacerbated muscle fatiguability. Taken together, these results demonstrate a novel role of AHR activation in muscle mitochondrial impairments caused by cigarette smoking.

While there is a wealth of literature documenting the alterations in skeletal muscle structure and function in patients with COPD,^6^ there is less understood about the direct effects of tobacco smoking on skeletal muscle physiology. Herein, 16‐weeks of cigarette smoke exposure did not produce changes in the muscle mass in the majority of hindlimb skeletal muscles (Figure 2D). The exception to this was the soleus muscle which displayed significant atrophy compared to air controls consistent with our previous study.^11^ In non‐COPD smokers, some data suggest greater propensity for myofibre atrophy in oxidative fibre types^20^ whereas others do not.^9^ Interpretation in COPD patients is complicated because many patients exhibit a shift away from pure type I fibres toward more hybrid fibres that express more than one myosin heavy chain, and thus atrophy may occur in the oxidative fibre types in COPD patients but that this fibre type shift may obscure this impact.

Interestingly, contractile function was unaffected by chronic cigarette smoke exposure in the current study (Figure 3). The lack of muscle weakness in the EDL muscle of smoke exposed mice is consistent with a previous study that reported normal EDL force production following 6 months of cigarette smoke exposure in C57BL6 mice.^21^ Similarly, daily cigarette smoke exposure did not exacerbate muscle fatigue in the EDL muscle of male mice is consistent with previous findings.^22^ In addition to measuring the EDL muscle, Rinaldi et al.^21^ also performed measures using the slow twitch soleus muscle where they reported significant decreases in specific force and elevated fatiguability. The latter study agrees with the findings of Nogueira et al.^23^ who reported that 8 weeks of nose cone‐based cigarette smoke exposure increase fatiguability of the plantarflexor muscles *in situ*. Unfortunately, we were unable perform muscle testing in multiple muscle groups due to the *in situ* approach used.

Mitochondrial alterations in muscle from patients with COPD include deficiency of oxidative enzymes,^3^ reduced mitochondrial density,^4^ and lower respiration rates.^5^, ^24^ These findings are consistent with *in vivo* measures of muscle oxidative capacity.^8^, ^25^ Employing rodent models of daily cigarette smoke exposure, similar alterations to COPD patients have been reported including decreases in citrate synthase activity,^10^, ^26^ reduced mitochondrial respiration rates,^11^, ^27^ and lower ATP synthesis rates.^18^ However, a recent study reported that 8‐months of cigarette smoking did not impair skeletal muscle ADP‐stimulated mitochondrial respiration in mice.^28^ The discrepancy in these studies may be related to experimental approaches employed to study mitochondrial function. In the current study, we employed experimental approaches that utilized either isolated skeletal muscle mitochondria or permeabilized myofibre bundles. Using isolated mitochondria, mitochondrial OXPHOS was significantly decreased by cigarette smoke exposure in both male and female mice. In contrast, the respiration rates normalized to the weight of the permeabilized myofibre bundles tended to be higher in mice exposed to cigarette smoke, especially in female mice. These results can be explained by a compensatory increase in mitochondrial biogenesis evidenced by increased citrate synthase enzyme activity in gastrocnemius muscle from smoke exposed mice.

A novel discovery from this study was that deletion of the AHR in skeletal muscle resulted in a significant increase in mitochondrial OXPHOS function in male, but not female mice. Interestingly, this group effect was observed in both air control and cigarette smoke exposed male mice. The AHR is a basic helix–loop–helix (bHLH) and Per‐Arnt‐Sim motifs transcription factor that is localized to the cytosol until ligand binding results in nuclear translocation where the AHR forms a dimer with the aryl hydrocarbon receptor nuclear translocator (ARNT) to facilitate DNA binding to xenobiotic response elements.^29^ While the role of the AHR in mediating acute responses to toxin exposure have been explored in numerous cell/tissue types, only a limited amount of data has examined a role in altering mitochondrial functions. For example, mitochondrial H_2_O_2_ production in liver was found to increase upon dioxin injection in wildtype but not AHR deficient mice.^13^ In spermatozoa, dioxin treatment caused depolarization of mitochondria in wildtype mice but not in AHR knockout mice.^30^ Employing proteomics, there is evidence that a portion of the AHR pool is localized to the mitochondrion and reported interactions with ATP5a1, an ATP synthase subunit.^14^ Hwang et al. further characterized AHR‐dependent mitochondrial proteome changes induced by dioxin treatment and revealed that AHR localizes to the intermembrane space and requires TOMM20 for import.^15^ In zebrafish, AHR activation was linked to mitochondrial ROS production and apoptosis following benzo[a]pyrene treatment.^31^

In agreement with previous work,^11^ the current study demonstrated that expression of a CAAHR in skeletal muscle compromised mitochondrial OXPHOS function (Figure 6). AAV9‐HSA‐CAAHR expression resulted in increased expression of pyruvate dehydrogenase kinases (*Pdk1* and *Pdk4*), which negatively regulate pyruvate dehydrogenase activity. Other changes included decreased levels of the *Ndufaf6*, *Ckm*, and *Vdac2*, as well as increased levels of *Ucp3*, *Aldh2*, *Polg*, and *Gsr*, which are indicative of mitochondrial stress. In AHR^mKO^ mice exposed to cigarette smoke, these alterations were abolished. Interestingly, *Polg* was altered in accordance with AHR activation levels, a finding that may be consistent with the high levels of mtDNA damage in COPD patient muscle.^32^ The oestrogen receptor (*Esr1*), whose expression was suppressed in CAAHR muscles but increased in AHR^mKO^ muscles has been linked to mitochondrial function in adipocytes, skeletal muscle, and pancreatic beta cells.^33^, ^34^, ^35^ In skeletal muscle, knockout of *Esr1* impairs mitochondrial respiration, increases H_2_O_2_ production, and alters mitochondrial morphology.^35^ The AHR/ARNT heterodimer can be physically associated with the oestrogen receptor alpha and regulate its transcriptional activity.^36^ A non‐canonical role of the AHR in promoting substrate delivery to the cullin 4B ubiquitin ligase to facilitate proteasomal degradation of the oestrogen receptor alpha has also been reported.^37^ Regardless of the mechanism by which AHR activation impairs OXPHOS in skeletal muscle, it is noteworthy to mention that the effects observed herein did not manifest in muscle atrophy or contractile dysfunction. Future studies are needed to explore if AHR‐mediated mitochondrial abnormalities precede the onset of muscle pathology such as that observed in models of cancer cachexia.^38^, ^39^

In contrast with a previous study,^11^ muscle‐specific expression of CAAHR did not reduce muscle mass herein. There are some experimental differences that could explain the incongruent findings. First, the previous study^11^ drove expression of the CAAHR using the cytomegalovirus (CMV) promoter and a hybrid AAV capsid (DJ) that can infect a broad range of cell types. In contrast, the muscle‐trophic AAV9 capsid was coupled with a human skeletal actin promoter to precisely drive CAAHR expression in mature myofibres herein, although it should be noted that AAV9 expression has been shown to have mosaicism with a preference for fast fibres.^40^ Emerging technologies have increased our understanding of the complex intercellular communication that occur between myofibres and resident interstitial cells. The fibro‐adipogenic progenitor cell has been linked to muscle atrophy through the paracrine release of cytokines.^41^ Another pathway for intercellular communication involves extracellular vesicles, which can contribute to tumour‐driven muscle atrophy.^42^ Thus, it is probable that AAV_DJ_‐CMV‐CAAHR resulted in AHR activation in non‐muscle cells that promote atrophy via unknown intercellular communication.

In conclusion, this study established that AHR activation in skeletal muscle promotes deficiencies in mitochondrial bioenergetics in male, but not female mice. Skeletal‐muscle specific deletion of the AHR significantly attenuated mitochondrial defects in male mice subjected to cigarette smoke.

## Conflict of interest

None declared.

## Funding

This research was funded by the James and Esther King Biomedical Research Program (Florida Department of Health), grant number 20K05 awarded to T.E.R. and R.T.H. L.F.F. was supported by a postdoctoral fellowship from the American Heart Association, grant number POST836216. T.T. was supported by a Ruth L. Kirschstein National Research Service Award Fellowship from the NIH/NIDDK, grant number F31‐DK128920. K.K. was supported by a postdoctoral fellowship from the American Heart Association, grant number POST903198.

## Supporting information


**Figure S1.** Muscle‐specific deletion of AHR does not impact mitochondrial hydrogen peroxide emission. (A) A description of the substrate protocol for isolated muscle mitochondrial hydrogen peroxide analysis. (B) Mitochondrial hydrogen peroxide emission (*J*H_2_O_2_) in isolated mitochondrial from skeletal muscle (gastrocnemius) in male and female mice, as well as quantification of *J*H_2_O_2_ under state 2 conditions. (C) Mitochondrial *J*H_2_O_2_ protocol performed in permeabilized myofiber bundles prepared from the red gastrocnemius muscle of mice. (D) Mitochondrial *J*H_2_O_2_ in permeabilized bundles across each step in the protocolAnalysis in all panels was done using two‐way ANOVA Šidák's post‐hoc testing for multiple comparisons when appropriate.
**Figure S2.** Uncropped western blot membranes for female mice. Uncropped images for western blotting analysis of mitochondrial OXPHOS protein complex abundance in female mice exposed to air or cigarette smoke. Quantification of band densitometry is shown in Figure 4.
**Figure S3.** AAV9‐GFP drives a robust immune response in skeletal muscle. (A) Volcano plot of mRNA levels as determined by RNA sequencing. Blue dots represent genes upregulated in AAV‐CAAHR treated mice. Orange dots represent genes downregulated in AAV‐CAAHR mice (B) Gene ontology analysis of significantly downregulated genes in AAV‐CAAHR treat mice indicate immune changes in the muscle. (C) Quantitative PCR analysis of selected immune genes from the RNA sequencing results demonstrates that these genes are not downregulated in AAV‐CAAHR treated mice when compared to naiive mice that were not infected with AAV. These analyses indicate the AAV‐GFP treatment promotes a local immune response in muscle. Error bars represent the standard deviation. Analysis in panel C was done using one‐way ANOVA.
