# Supplementary material for: Chronic aryl hydrocarbon receptor activity impairs muscle mitochondrial function with tobacco smoking
Source: J Cachexia Sarcopenia Muscle. 2024 Feb 9;15(2):646–59. doi: 10.1002/jcsm.13439 (PMC10995249; doi:10.1002/jcsm.13439)
Supplement: Supplementary file 1 — Figure S1. Muscle‐specific deletion of AHR does not impact mitochondrial hydrogen peroxide emission. (A) A description of the substrate protocol for isolated muscle mitochondrial hydrogen peroxide analysis. (B) Mitochondrial hydrogen peroxide emission (JH2O2) in isolated mitochondrial from skeletal muscle (gastrocnemius) in male and female mice, as well as quantification of JH2O2 under state 2 conditions. (C) Mitochondrial JH2O2 protocol performed in permeabilized myofiber bundles prepared from the red gastrocnemius muscle of mice. (D) Mitochondrial JH2O2 in permeabilized bundles across each step in the protocolAnalysis in all panels was done using two‐way ANOVA Šidák's post‐hoc testing for multiple comparisons when appropriate. Figure S2. Uncropped western blot membranes for female mice. Uncropped images for western blotting analysis of mitochondrial OXPHOS protein complex abundance in female mice exposed to air or cigarette smoke. Quantification of band densitometry is shown in Figure 4. Figure S3. AAV9‐GFP drives a robust immune response in skeletal muscle. (A) Volcano plot of mRNA levels as determined by RNA sequencing. Blue dots represent genes upregulated in AAV‐CAAHR treated mice. Orange dots represent genes downregulated in AAV‐CAAHR mice (B) Gene ontology analysis of significantly downregulated genes in AAV‐CAAHR treat mice indicate immune changes in the muscle. (C) Quantitative PCR analysis of selected immune genes from the RNA sequencing results demonstrates that these genes are not downregulated in AAV‐CAAHR treated mice when compared to naiive mice that were not infected with AAV. These analyses indicate the AAV‐GFP treatment promotes a local immune response in muscle. Error bars represent the standard deviation. Analysis in panel C was done using one‐way ANOVA. [file JCSM-15-646-s001.docx]

**SUPPLEMENTAL MATERIAL**

**Chronic aryl hydrocarbon receptor activation impairs muscle mitochondrial function with tobacco smoking**

Liam F. Fitzgerald^2,*^, Jacob Lackey^1,*^, Ahmad Moussa^1^, Sohan V. Shah^2^, Ana Maria Castellanos^1^, Shawn Khan^1^, Martin Schonk^2^, Trace Thome^1^, Zachary R. Salyers^1^, Niska Jakkidi^1^, Kyoungrae Kim^1^, Qingping Yang^1^, Russell T. Hepple^2,4,*^ Terence E. Ryan^1,3,4,*,#^,

^1^Department of Applied Physiology and Kinesiology, ^2^Department of Physical Therapy, ^3^Center for Exercise Science, ^4^Myology Institute, University of Florida, Gainesville, FL, USA

**Running Head:** Role of the AHR in smoking-induce muscle pathology

**^*^**These authors contributed equally.

**^#^Correspondence:** Terence E. Ryan, PhD: 1864 Stadium Rd, Gainesville, FL, 32611. Tel: 352-294-1700 (office); email: [ryant@ufl.edu](mailto:ryant@ufl.edu); Twitter: @TerenceRyan_PhD

**KEYWORDS:** cigarette, atrophy, skeletal muscle, dioxin

**Funding:** This research was funded by the James and Esther King Biomedical Research Program (Florida Department of Health), grant number 20K05 awarded to T.E.R. and R.T.H. L.F.F. was supported by a postdoctoral fellowship from the American Heart Association, grant number POST836216. T.T. was supported by a Ruth L. Kirschstein National Research Service Award Fellowship from the NIH/NIDDK, grant number F31-DK128920. K.K. was supported by a postdoctoral fellowship from the American Heart Association, grant number POST903198.

**Disclosures:** None.

**Expanded Materials and Methods**

**Animals*.*** We generated muscle-specific AHR knockout mice (AHR^mKO^) by breeding conditionally floxed AHR mice (AHR^tm3.1Bra^/J, Jackson Laboratories, Stock No. 006203) with HSA-MCM mice that express MerCreMer double fusion protein under the control of the human ACTA1 (actin, alpha 1, skeletal muscle) promoter (Tg(ACTA1-cre/Esr1*)2Kesr/J, Jackson Laboratories, Stock No. 025750). Muscle-specific AHR knockout was induced by intraperitoneal injection of tamoxifen (80mg/kg) for five consecutive days. Muscle-specific DNA recombination was confirmed using PCR amplification of genomic DNA using primers flanking Exon 2 (Forward = 5-atcttgtgtcaggaacaggccatc-3’ and Reverse = 5’-ggtacaagtgcacatgcctgc-3’). For experiments involving AAV delivery, five-month-old C57BL6J mice (n=48) were purchased from Jackson Laboratories (Stock No. 000664). For experiments assessing temporal changes in AHR signaling following acute cigarette smoke exposure, three and a half-month-old C57BL6J male mice (n=32) were purchased from Jackson Laboratories (Stock No. 000664). All mice were housed in a temperature- (22°C) and light-controlled (12h light/12h dark) room and maintained on standard chow diet (Envigo Teklad Global 18% Protein Rodent Diet 2918 irradiated pellet) with free access to food and water. All animal experiments adhered to the Guide for the Care and Use of Laboratory Animals from the Institute for Laboratory Animal Research, National Research Council, Washington, D.C., National Academy Press, 2011, and any updates. All procedures were approved by the Institutional Animal Care and Use Committee of the University of Florida (Protocol 202009766).

**Cigarette Smoke Exposure*.*** Four weeks following the last intraperitoneal injection of tamoxifen, AHR^mko^ mice were exposed to either room air or tobacco smoke (TS) for 16 weeks, with TS exposures as described previously [1, 2]. Briefly, 3R4F research-grade cigarettes were purchased from the University of Kentucky (Lexington, KY, USA) and smoked according to the protocol approved by the Federal Trade Commission (1 puff per minute per cigarette, where each puff was 2 s in duration and 35 ml in volume) using a Teague TE-10 whole-body smoke exposure system (Teague Enterprises, Woodland, CA, USA). Mice in the TS group were exposed for 1 h, twice per day, 5 days per week for a total of 16 weeks. The concentration of TS was measured during each smoke exposure using a standard flow meter and filter paper per manufacturer protocol. The average TS concentration across the 16 weeks was 220±80mg/m^3^. Mice in the air-exposed group were kept in the same barrier facility as the TS-exposed mice, but not exposed to TS.

**AAV Construction and Delivery*.*** To accomplish muscle cell-specific overexpression of transgenes, the human skeletal actin (ACTA1; termed HSA herein) promoter (1541 bp proximal to the transcription start site) was PCR amplified from human genomic DNA isolated from a donor muscle biopsy. The AAV-HSA-GFP plasmid was developed by inserting a human HSA promoter and GFP (ZsGreen1) into the promoterless AAV vector (Cell BioLabs, Cat. No. VPK-411-DJ) using In-Fusion Cloning (Takara Bio, Cat. No. 638911). Similarly, the mouse AHR, including ligand binding domain, was PCR amplified from cDNA obtained from a C57BL6J mouse and inserted downstream of the HSA promoter. To generate a constitutively active AHR (CAAHR) vector, the mouse AHR coding sequence was PCR amplified from cDNA obtained from a C57BL6J mouse such that the ligand binding domain (amino acids 277–418) was deleted, and subsequently inserted downstream of the HSA promoter. The resulting plasmids were packaged using AAV2/9 serotype by Vector Biolabs (Malvern, PA). AAV9 was delivered via intramuscular injections of the hind limb (gastrocnemius, tibialis anterior (TA), extensor digitorum longus (EDL)) muscles of both legs at a dosage of 5E+11 vg/limb.

**Preparation of Permeabilized Myofiber Bundles*.*** Mice were anaesthetized by intraperitoneal injection of ketamine (90 mg/kg) and xylazine (10 mg/kg) and the gastrocnemii removed, blotted to remove excess blood, and weighed. The red portions

of the gastrocnemii were then carefully dissected using a pair of sharp scissors and then placed into pre-cooled buffer A (CaK_2_EGTA (2.77mM), K_2_EGTA (7.23mM), MgCl_2_ (6.56mM), dithiothreitol (0.5mM), K-MES (50mM), imidazole (20mM), taurine (20mM), Na_2_ATP (5.3mM), phosphocreatine (15mM), pH 7.3 at 4°C). The remaining portions of the gastrocnemii were either used to isolate skeletal muscle mitochondria (see below) or snap-frozen in liquid nitrogen and stored at -80°C for subsequent analysis. Thin fiber bundles from the red gastrocnemii were carefully separated along their fiber orientation in buffer A at 4°C, as described previously [3]. Myofiber bundles were then permeabilized by mild shaking for 30 min in buffer A supplemented with saponin (50µg/ml). After permeabilization, fiber bundles were immediately washed for 10 min in buffer D (MgCl_2_-6H_2_O (5mM), K-MES (105mM), KCl (30mM), KH_2_PO_4_ (10mM), EGTA (1mM), BSA (2.5g/L), pH 7.2 at room temperature), then blotted dry to remove excess water and weighed prior to measurement of oxygen consumption or H_2_O_2_ emission.

**Isolation of Skeletal Muscle Mitochondria.** To isolate skeletal muscle mitochondria, the gastrocnemius muscle was rapidly dissected and placed in ice-cold PBS supplemented with 10mM EDTA. The muscle was carefully trimmed of fat and connect tissues, minced on ice, and digested for five-minute with 0.025% w/v trypsin (Millipore-Sigma, Cat. No. T4799). Following trypsin digestion, the tissue was centrifuged for five minutes at 200xG and the supernatant was aspirated. The digested tissue pellet was resuspended in Buffer C (MOPS (50mM), KCl (100mM), EGTA (1mM), MgSO_4_ (5mM), bovine serum albumin (BSA; 2g/L); pH=7.1) and then homogenized via a glass-Teflon homogenizer (Wheaton) and subsequently centrifuged at 800xG for ten minutes. The tissue pellet was discarded, and the resulting supernatant was centrifuged at 10,000xG for ten minutes to pellet mitochondria. All steps were performed at 4°C. The mitochondrial pellet was gently washed to remove any damaged mitochondria and then re-suspended in Buffer B (MOPS (50mM), KCl (100mM), EGTA (1mM), MgSO_4_ (5mM); pH=7.1) and protein concentration was determined using bicinchoninic acid protein assay (ThermoFisher Scientific, Cat. No. A53225).

**Assessment of Mitochondrial Oxygen Consumption and Hydrogen Peroxide Emission*.*** For experiments involving isolated mitochondria, respiratory function was assessed at 37°C in buffer D (in mmol/l) supplemented with creatine monohydrate (5 mM), using the OROBOROS O2K Oxygraph. Isolated mitochondria were energized with glutamate and malate (10mM and 2.5mM respectively) to obtain state 2 respiration, followed by

addition of 20U/ml creatine kinase, 5mM ATP, and 1mM phosphocreatine (PCr) to mimic a near maximal exercise condition. Subsequent additions of PCr were added stepwise to bring the cellular energy demand down to resting conditions. The slope of the relationship between cellular energy demand (∆G_ATP_) and oxygen consumption (*J*O_2_) was calculated (termed OXPHOS conductance)[4]. The rate of respiration was expressed as pmol/sec/mg of mitochondria. All respiration measurements were conducted at 37°C and a working range [O_2_] of ~200 μM. H_2_O_2_ production was assessed using identical substrate conditions as performed in the OROBOROS O2K Oxygraph via the Amplex Ultra Red/horseradish peroxidase detection system as previously described [5, 6].

For experiments involving permeabilized myofiber bundles, mitochondrial respiratory capacity was assessed at 37°C in buffer D supplemented with creatine monohydrate (5

mM) using a polarographic oxygen sensor (Oxygraph-2k; Oroboros, Innsbruck, Austria). Briefly, small 1-2 mg (wet weight) permeabilized bundles were added to the respiration chambers (one bundle per chamber). Myofiber respiration was performed at [O_2_] ≥150 μM to eliminate O_2_ diffusion limitations. Mitochondria were energized with glutamate and malate (10 mM and 5 mM, respectively) to obtain state 2 respiration of CI, adenosine diphosphate (ADP, 2 mM) to obtain state 3 respiration of CI, and succinate (10 mM) to obtain CI+CII-driven state 3 respiration. Next, cytochrome c (10 μM) was added to the chambers to assess the integrity of the outer mitochondrial membrane. Experiments with a ≥10% increase in respiration upon addition of cytochrome c were not used for analysis. To determine the respiratory capacity of CIV, independent of upstream complexes, antimycin A (10 μM) was added to the chambers, followed by ascorbate and N,N,N’,N’-tetramethyl-p-phenylenediamine (TMPD; 10 mM and 1 mM,

respectively). To evaluate the OXPHOS coupling efficiency, we calculated the acceptor control ratio (state 3:2). The rate of respiration was expressed as pmol/sec/mg of muscle tissue (wet weight).

H_2_O_2_ production was assessed in permeabilized myofiber bundles using a spectrofluorometer (FluoroMax, Horiba Scientific Instruments, Irvine, CA, USA) with the following parameters (Excitation: 565nm; Emission: 600nm; 2nm slit widths). Mitochondrial ROS production was measured based on H_2_O_2_ emission detected by the reaction of H_2_O_2_ with Amplex UltraRed, catalyzed by horseradish peroxidase, as described previously [7, 8]. Small (1-2 mg wet weight) permeabilized bundles were

added to a quartz cuvette containing 1mL buffer D supplemented with creatine monohydrate (5 mM). After basal H2O2 emission was determined, mitochondria were energized with glutamate and malate (10mM and 5mM, respectively), succinate (10mM), sub-maximal ADP (0.1mM), maximal ADP (2mM), and lastly antimycin A (10 μM). To allow calculation of H_2_O_2_ production rates, a standard calibration curve was generated using titrations of a known concentration of H_2_O_2_. All experiments were performed at 37°C.

**Nerve-mediated Muscle Contractile Function.** Skeletal muscle contractile function was assessed in the EDL muscle *in-situ* using stimulation of the peroneal nerve. Mice were anesthetized with ketamine (90mg/kg) and xylazine (10mg/kg) and the distal EDL tendon was carefully isolated, and a silk ligature was tied and attached to the lever arm of the force transducer (Cambridge Technology; Model: 2250). The hindlimb was stabilized to prevent movement and temperature of the platform maintained at 37°C using a water bath. Needle electrodes (Chalgren, Cat. No. 111-725-24TP) were inserted on either side of the peroneal nerve and attached to a 701A stimulator (Aurora Scientific) and 0.2ms pulses at 15V were delivered. Data collection and servomotor control were manipulated using a Lab-View-based DMC program (version v5.500). Optimal length was determined by applying twitch contractions and adjusting the muscle length. Force frequency curves were created by stimulating at 1, 25, 50, 75, 100, 125, 150, and 175 Hz with one-minute rest between contractions. Following force frequency testing, muscle fatiguability was assessed by delivering 50Hz contractions every two seconds for three minutes. Recovery from fatigue was examined by delivering additional 50Hz contractions at 1, 3, 5, and 10-minutes post-fatigue testing. Specific force was calculated by normalizing forces to the muscle weight.

**Skeletal Muscle Histology and Immunofluorescence Microscopy*.*** 10-µm-thick transverse sections of the TA, EDL, and Soleus (Sol) muscles were cut using a cryotome (Leica CM3050S) and collected on slides for staining. Skeletal myofiber cross-sectional area (CSA) was assessed by staining muscle sections with 5μg/ml wheat germ agglutinin conjugated to AlexaFluor-647 (Invitrogen, Cat. No. W32466), washed with PBS, and cover slipped with Vectashield Hardmount with DAPI (Vector Laboratories, Cat. No. H-1500). Images were obtained at 20x magnification using an Evos FL2 Auto microscope (ThermoFisher Scientific) and tiled/merged images of the entire muscle cross-section were used for analysis. Myofiber CSA analysis was performed using MuscleJ [9], an automated image analysis program in Fiji.

**RNA-isolation and qRT-PCR*.*** Total RNA was extracted from gastrocnemius muscle using TRIzol (Invitrogen, Cat. No.15-596-018). The muscle sample was homogenized with TRIzol using PowerLyzer 24 (Qiagen), and RNA was isolated using Direct-zol RNA MiniPrep kit (Zymo Research, R2052) following the manufacturer’s direction. cDNA was generated from 500ng of RNA using the LunaScript RT Supermix kit (New England Biolabs, E3010L) according to the manufacturer’s directions. Real-time PCR (RT-PCR) was performed on a Quantstudio 3 (ThermoFisher Scientific) using Luna Universal qPCR master mix (New England Biolabs, M3003X) and the following primers: AHR (Forward-AACATCACCTATGCCAGCCG, Reverse-GGTCTCTGTGTCGCTTAGAAGG), Cyp1A1 (Forward-CAGCCTTCCCAAATGGTTTA, Reverse-GCCTGGGCTACACAAGACTC), and L32 (Forward-TTCCTGGTCCACAATGTCAA, Reverse-GGCTTTTCGGTTCTTAGAGGA) was used as the housekeeping control. Relative gene expression was calculated using 2^-ΔΔCT^ from the relevant control group.

**Immunoblotting.** Protein abundance was measured using western blotting approaches as follows. Muscles were lysed in CelLytic M (Millipore-Sigma, Cat. No. C2978) and protein concentrations determined by bicinchoninic acid protein assay (ThermoFisher Scientific, Cat No. A53225). Lysates were mixed with Laemmli sample buffer (Bio-Rad, Cat. No. 1610737) supplemented with beta-mercaptoethanol and proteins were separated using SDS-PAGE gels (Bio-Rad, Cat. Nos. 5678044 or 4561094) and subsequently transferred to polyvinylidene fluoride (PVDF) membranes. Total protein on the PVDF membrane was quantified using Bio-Rad’s Stain Free technology. PVDF membranes were then blocked for 90 minutes in blocking buffer (Licor, Cat. No. 927-60001). To examine protein abundance of mitochondrial electron transport system complexes, the PVDF membranes were incubated overnight with an antibody cocktail targeting protein subunit of each electron transport system complex (Abcam, Cat. No. ab110413; 1:1000 dilution). The following morning, membranes were washed with tris-buffered saline containing 0.1% (v/v) Tween-20 and then incubated with appropriate secondary antibodies conjugated to IRDye 680RD from Licor (1:15,000 dilution). Images were acquired using a Licor Odyssey DLx and band intensities were quantified using Licor’s Empiria Studio software.

**Citrate Synthase Activity Assay.** Gastrocnemius muscles were lysed in CelLytic M (Millipore-Sigma, Cat. No. C2978) and protein concentrations determined by bicinchoninic acid protein assay (ThermoFisher Scientific, Cat No. A53225). Twenty micrograms of lysate were loaded into a 96-well plate and citrate synthase activity was measured using a commercially available kit (Millipore-Sigma, Cat. No. CS0720).

**Gene expression profiling in human muscle specimens.** To examine the AHR signaling pathway in human muscle specimens from patients with chronic obstructive pulmonary disease (COPD) and age-matched healthy controls that were non-smokers, we analyzed a publicly available microarray dataset (GEO100281). Information on the

physical and clinical characteristics of patients, as well as sample processing can be found in the original publication [10]. Gene expression data were RMA-treated using Affymetrix Power Tools (APT, 1.16.1) and imported into R (version 4.2.0).

**RNA sequencing in mouse muscle specimens.** Total RNA was extracted from gastrocnemius muscle using the Direct-zol RNA MiniPrep kit (Zymo Research, Cat. No. R2052) following the manufacturer’s direction. Library preparation and mRNA sequencing via PolyA selection was performed by Genewiz (Azenta Life Science, South Plainfield, NJ). Paired-end 150bp reads were sequenced on an Illumina HiSeq 4000. Sequence reads were trimmed to remove possible adapter sequences and nucleotides with poor quality using Trimmomatic v.0.36. The trimmed reads were mapped to the Mus musculus GRCm38 reference genome available on ENSEMBL using the STAR aligner v.2.5.2b. Unique gene hit counts were calculated by using featureCounts from the Subread package v.1.5.2. The hit counts were summarized and reported using the gene_id feature in the annotation file. Only unique reads that fell within exon regions were counted. If a strand-specific library preparation was performed, the reads were strand-specifically counted. After extraction of gene hit counts, the gene hit counts table was used for downstream differential expression analysis. Using DESeq2, a comparison of gene expression between the customer-defined groups of samples was performed. The Wald test was used to generate p-values and log2 fold changes. Genes with an adjusted p-value < 0.05 and absolute log2 fold change > 1 were called as differentially expressed genes for each comparison. Below are the results of the number of significantly differentially expressed genes for all comparisons provided. A gene ontology analysis was performed on the statistically significant set of genes by implementing the software GeneSCF v.1.1-p2. The mgi GO list was used to cluster the set of genes based on their biological processes and determine their statistical significance. A list of genes clustered based on their gene ontologies was generated. The raw data have been deposited in NCBI’s Gene Expression Omnibus and can be accessed using accession numbers GSE225607 and GSE225670.

**Statistical analysis*.*** Data are presented as mean ± SD. Normality of data was tested with the Shapiro-Wilk test. Comparisons between two groups were performed by Student’s unpaired two-tailed *t*-test. If variances were found to be different between groups, an unpaired *t*-test with Welch’s correction was performed. Comparisons of data with more than two groups were performed using two-way ANOVA with Šidák’s post-hoc testing for multiple comparisons when significant interactions were detected. All statistical analysis was performed in GraphPad Prism (Version 9.0). In all cases, *P*<0.05 was considered statistically significant.


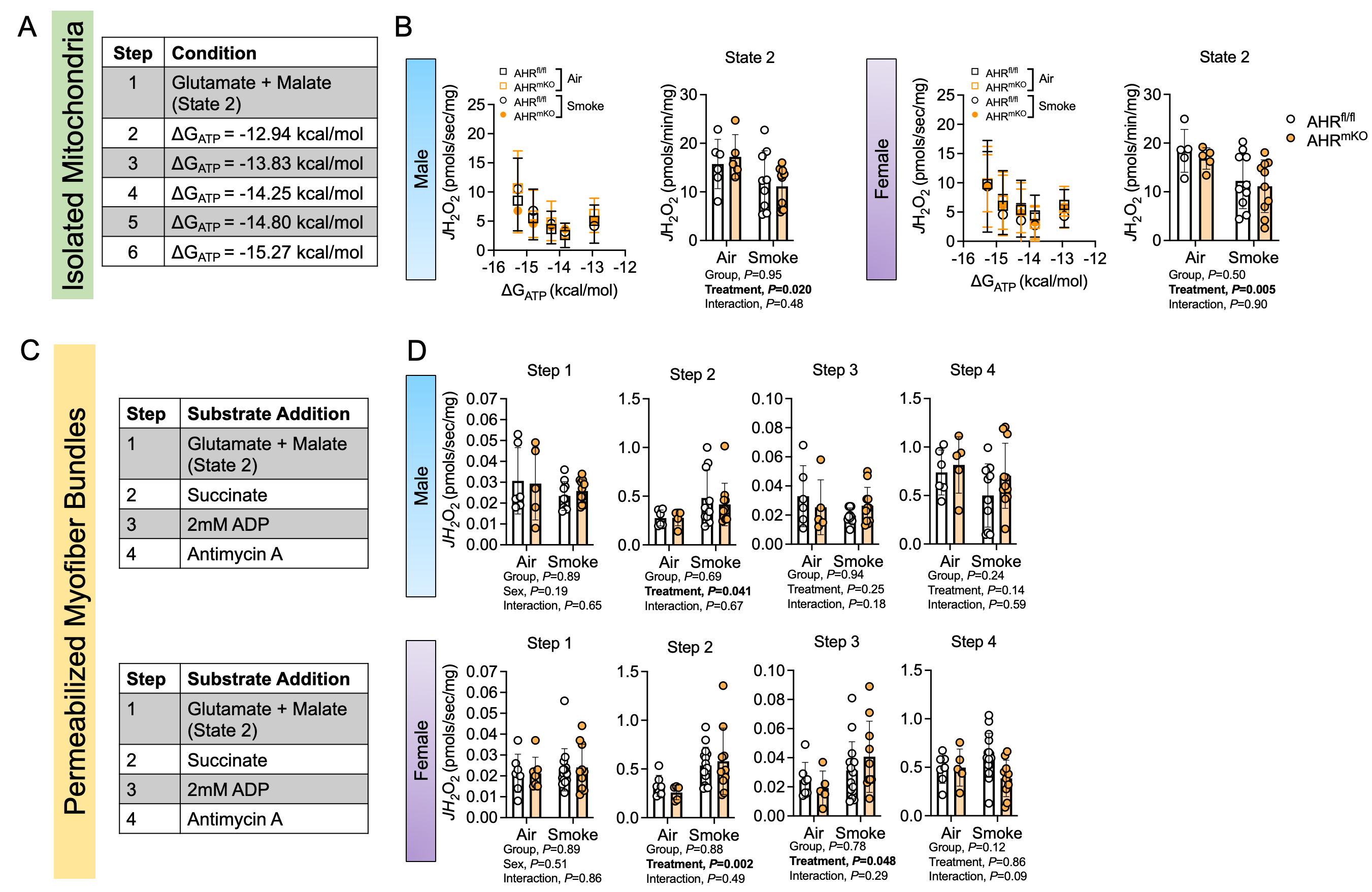


**Supplemental Figure 1. Muscle-specific deletion of AHR does not impact mitochondrial hydrogen peroxide emission.** (**A**) A description of the substrate protocol for isolated muscle mitochondrial hydrogen peroxide analysis. (**B**) Mitochondrial hydrogen peroxide emission (*J*H_2_O_2_) in isolated mitochondrial from skeletal muscle (gastrocnemius) in male and female mice, as well as quantification of *J*H_2_O_2_ under state 2 conditions. (**C**) Mitochondrial *J*H_2_O_2_ protocol performed in permeabilized myofiber bundles prepared from the red gastrocnemius muscle of mice. (**D**) Mitochondrial *J*H_2_O_2_ in permeabilized bundles across each step in the protocolAnalysis in all panels was done using two-way ANOVA Šidák’s post-hoc testing for multiple comparisons when appropriate.


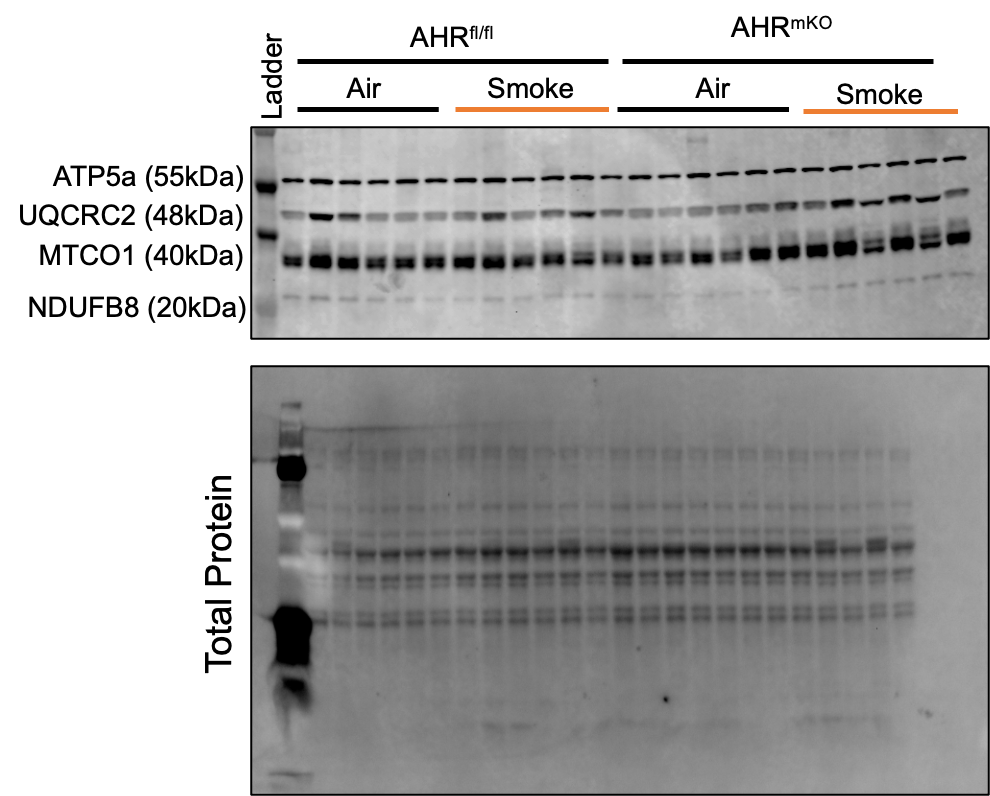


**Supplemental Figure 3. Uncropped western blot membranes for female mice.** Uncropped images for western blotting analysis of mitochondrial OXPHOS protein complex abundance in female mice exposed to air or cigarette smoke. Quantification of band densitometry is shown in Figure 4.


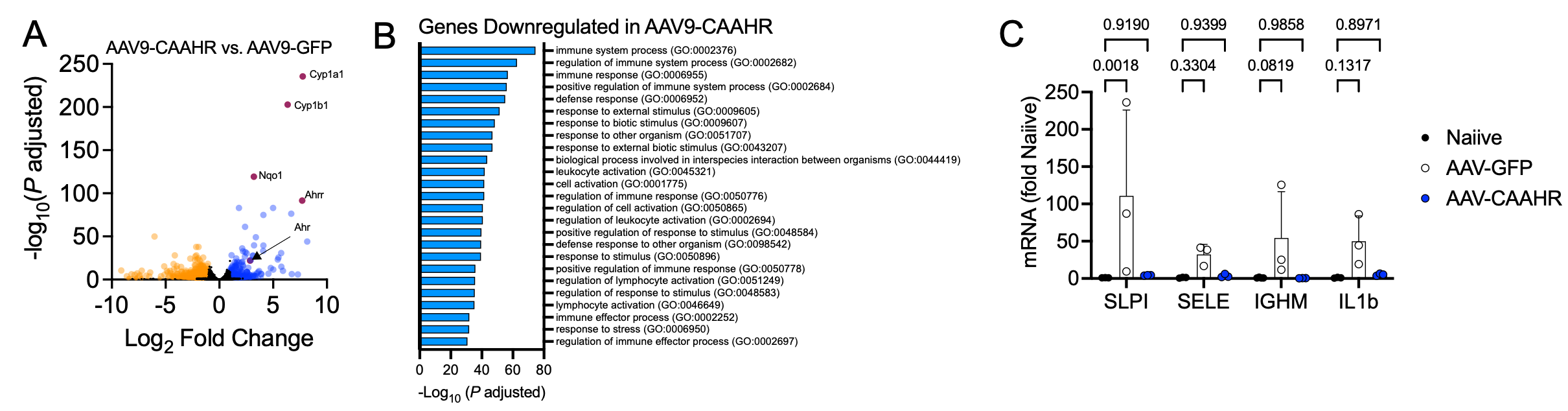


**Supplemental Figure 3. AAV9-GFP drives a robust immune response in skeletal muscle.** (**A**) Volcano plot of mRNA levels as determined by RNA sequencing. Blue dots represent genes upregulated in AAV-CAAHR treated mice. Orange dots represent genes downregulated in AAV-CAAHR mice (**B**) Gene ontology analysis of significantly downregulated genes in AAV-CAAHR treat mice indicate immune changes in the muscle. (**C**) Quantitative PCR analysis of selected immune genes from the RNA sequencing results demonstrates that these genes are not downregulated in AAV-CAAHR treated mice when compared to naiive mice that were not infected with AAV. These analyses indicate the AAV-GFP treatment promotes a local immune response in muscle. Error bars represent the standard deviation. Analysis in panel C was done using one-way ANOVA.

**Supplementary References**

1. de Souza, A.R., et al., *Aryl hydrocarbon receptor (AhR) attenuation of subchronic cigarette smoke-induced pulmonary neutrophilia is associated with retention of nuclear RelB and suppression of intercellular adhesion molecule-1 (ICAM-1).* Toxicological Sciences, 2014. **140**(1): p. 204-223.

2. Kapchinsky, S., et al., *Smoke‐induced neuromuscular junction degeneration precedes the fibre type shift and atrophy in chronic obstructive pulmonary disease.* The Journal of physiology, 2018. **596**(14): p. 2865-2881.

3. Picard, M., et al., *Resistance to Ca2+-induced opening of the permeability transition pore differs in mitochondria from glycolytic and oxidative muscles.* American Journal of Physiology-Regulatory, Integrative and Comparative Physiology, 2008. **295**(2): p. R659-R668.

4. Fisher-Wellman, K.H., et al., *Mitochondrial Diagnostics: A Multiplexed Assay Platform for Comprehensive Assessment of Mitochondrial Energy Fluxes.* Cell Rep, 2018. **24**(13): p. 3593-3606 e10.

5. Thome, T., M.D. Coleman, and T.E. Ryan, *Mitochondrial Bioenergetic and Proteomic Phenotyping Reveals Organ-Specific Consequences of Chronic Kidney Disease in Mice.* Cells, 2021. **10**(12).

6. Thome, T., et al., *Uremic metabolites impair skeletal muscle mitochondrial energetics through disruption of the electron transport system and matrix dehydrogenase activity.* American Journal of Physiology-Cell Physiology, 2019. **317**(4): p. C701-C713.

7. Picard, M., et al., *Mitochondrial functional impairment with aging is exaggerated in isolated mitochondria compared to permeabilized myofibers.* Aging cell, 2010. **9**(6): p. 1032-1046.

8. Anderson, E.J. and P.D. Neufer, *Type II skeletal myofibers possess unique properties that potentiate mitochondrial H2O2 generation.* American Journal of Physiology-Cell Physiology, 2006. **290**(3): p. C844-C851.

9. Mayeuf-Louchart, A., et al., *MuscleJ: a high-content analysis method to study skeletal muscle with a new Fiji tool.* Skelet Muscle, 2018. **8**(1): p. 25.

10. Willis-Owen, S.A.G., et al., *COPD is accompanied by coordinated transcriptional perturbation in the quadriceps affecting the mitochondria and extracellular matrix.* Scientific Reports, 2018. **8**.
